# A novel cloning strategy for one-step assembly of multiplex CRISPR vectors

**DOI:** 10.1038/s41598-018-35727-3

**Published:** 2018-11-30

**Authors:** Marc Zuckermann, Mario Hlevnjak, Haniyeh Yazdanparast, Marc Zapatka, David T. W. Jones, Peter Lichter, Jan Gronych

**Affiliations:** 1 Division of Molecular Genetics, German Cancer Research Center (DKFZ), Im Neuenheimer Feld 280, 69120 Heidelberg, Germany; 2 Division of Pediatric Neurooncology, German Cancer Research Center (DKFZ), Im Neuenheimer Feld 280, 69120 Heidelberg, Germany; 3 German Cancer Consortium (DKTK), Im Neuenheimer Feld 280, 69120 Heidelberg, Germany; 4 Hopp Children’s Cancer Center Heidelberg, 69120 Heidelberg, Germany

## Abstract

One key advantage of the CRISPR/Cas9 system in comparison with other gene editing approaches lies in its potential for multiplexing. Here, we describe an elaborate procedure that allows the assembly of multiple gRNA expression cassettes into a vector of choice within a single step, termed ASAP(Adaptable System for Assembly of multiplexed Plasmids)-cloning. We demonstrate the utility of ASAP-cloning for multiple CRISPR-mediated applications, including efficient multiplex gene editing, robust transcription activation and convenient analysis of Cas9 activity in the presence of multiple gRNAs.

## Introduction

The possibility of multiplexing is one of the advantageous features of the CRISPR/Cas9 system compared with other gene editing approaches (reviewed by Dominguez *et al*.^1^). Due to the increasing demand for multiplex gene targeting, e.g. when modeling complex diseases, there have been a number of reports on sophisticated cloning strategies to generate the required constructs. Besides techniques that adapted Gibson Assembly^2,3^, several methods that have been used for this purpose derive from Golden Gate cloning^4–9^, featuring multiple advantages but also limitations in comparison to Gibson Assembly-related methods. Golden Gate approaches are usually based on the construction of numerous entry plasmids containing individual DNA fragments that are ultimately used to reconstitute the desired insert. These fragments are flanked by type IIS restriction endonuclease (TIIS-RE) sites and unique single strand overhangs. By undergoing iterations of restriction and ligation, the fragments can be assembled seamlessly in a defined order and inserted into a specific destination vector within one reaction. However, these techniques suffer from the following limitations: the initial step of library construction is i) time-consuming and ii) does not allow for simple subtraction or addition of further inserts and thereby renders the procedure inflexible; iii) previously published techniques rely on specific destination vectors with TIIS-RE sites, making these methods only useful for a defined context.

Here, we report how these limitations can be overcome by a novel strategy advancing key aspects of the original Golden Gate cloning principle. To obviate the preparation of entry plasmids, we designed a “PCR-on-ligation” step for flexible generation of individual insert fragments. Furthermore, elaborate primer design and novel utilization of pairs of isocaudomers (type II restriction enzymes having slightly different recognition sites but producing identical overhangs) allows for ligation of the generated inserts into type II restriction endonuclease (TII-RE) sites in a large variety of common expression vectors, enabling utilization of this technique in a wide range of applications. We demonstrate this utility by constructing different multiplex CRISPR vectors for various downstream applications. Based on these characteristics, this method is termed “ASAP-cloning” (*A*daptable *S*ystem for *A*ssembly of multiplexed *P*lasmids).

## Results

**Figure 1:**
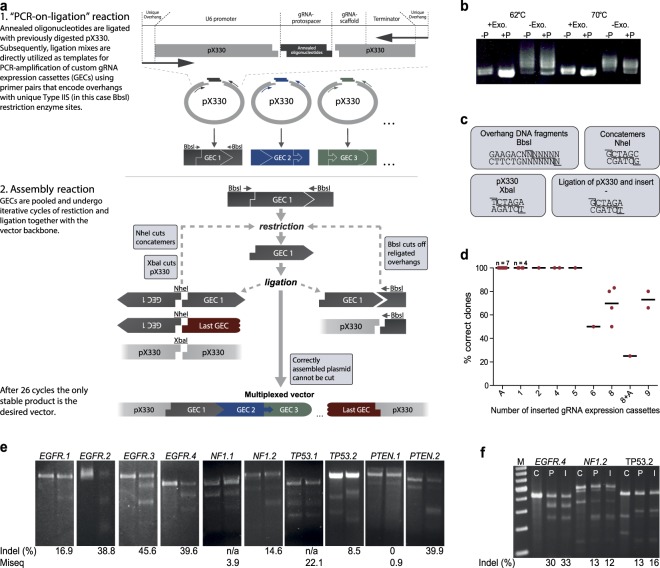
ASAP-cloning allows rapid vector generation for efficient multiplex gene targeting. (**a**) Workflow of ASAP-cloning. Annealed oligonucleotides, encoding the protospacer complementary region of the gRNA are ligated with the pX330 vector. Subsequently, gRNA expression cassettes (GECs), including the U6 promoter, the gRNA and a transcriptional terminator are amplified directly from the ligation mix. The utilized primers encode for unique restriction enzyme sites that are attached to the GEC during PCR-amplification. In the following assembly reaction, addition of distinct enzymes to cleave GEC overhangs allows to array the respective GECs in a defined order and to insert them into a vector of choice within one process. The only stable plasmid vacant of functional restriction sites during the reaction is the desired product, which is sufficiently enriched after 26 cycles of restriction and ligation. (**b**) Exonuclease treatment and the use of non-dephosphorylated backbones are crucial for the generation of distinct PCR products. After ligation of annealed oligodinucleotides with either dephosphorylated (−P) or non-dephosphorylated (+P), BbsI-cut pX330 vector, an exonuclease treatment was performed for half of the analyzed ligation mixes. Subsequently, reaction mixes of either the exonuclease treatment (+Exo.) or the ligation (−Exo.) were used as template for a PCR. Annealing temperatures of 62 °C or 70 °C were used, as indicated. (**c**) Restriction enzyme sites utilized in the construction of pX330-10x. The boxes indicate which DNA molecule was cut with which enzyme and the respective sequence. (**d**) Efficiency of ASAP-cloning depends on the number of GECs forming the combined insert. Each dot represents an independent cloning approach with the indicated, inserted number of GECs. Bars indicate mean values. Correctness of clones was analyzed *via* control restriction and Sanger sequencing. “A” indicates the addition of annealed oligonucleotides to the reaction mix. During ASAP-cloning these were inserted into the designated gRNA expression cassette of the original pX330 vector. (**e**) SURVEYOR assays to determine the frequency of generated indels at targeted loci. Each locus was amplified from control gDNA (left lane) and from gDNA isolated from cells that had been transfected with pX330-10x (right lane). Resulting PCR products were used in SURVEYOR assays. For some loci, control DNA was digested as well, indicating PCR artifacts or endogenous SNVs. In this case, the resulting bands were added to the “undigested” fraction when calculating indel frequencies. Loci for which the SURVEYOR assay did not yield measurable results were sequenced on the MiSeq platform. (**f**) pX330-10x and pX330-impsc-10x display similar genome editing efficiencies. SURVEYOR assays of the indicated PCR products were performed after isolation of gDNA from untransfected cells (C) or cells that had been transfected with either pX330-10x (P) or pX330-impsc-10x (I). M = GeneRuler 100 bp DNA Ladder.

To exemplify the workflow of ASAP-cloning we generated a CRISPR/ Cas9 vector (pX330) with multiple arrayed gRNA sequences as illustrated in Fig. 1a. As a first step, for each individual gRNA expression cassette (GEC), the “PCR-on-ligation” reaction is performed by ligating annealed oligonucleotides, encoding the protospacer complementary region of the gRNA, into the pX330 backbone according to the original Zhang lab protocol^10^, except that backbones are not dephosphorylated. This allows a nick-free ligation of the DNA backbone and the respective annealed oligonucleotides during the ligation step, generating intact circular plasmids. After incubation with an exonuclease to remove residual non-circular backbones or inserts, these ligation mixes are directly used as the input for subsequent PCRs without further processing. For the respective PCR, primer pairs flanking the entire GEC, comprising the U6 promoter, the gRNA and a transcriptional terminator, are designed. Additionally, on their 5´ends the primers contain specific TIIS-RE sites, analogous to the original Golden Gate protocol (Table 1). Importantly, the use of a phosphorylated backbone and the exonuclease reaction both proved to be crucial for generating specific PCR products from the ligation mix (Figs 1b and S1a). After purification, the PCR products are directly used as insert fragments for the subsequent assembly reaction.

**Table 1:** Suitable oligonucleotides for the amplification of gRNA expression cassettes.

Oligonucleotide	Sequence
ASAP_BbsI_fwd_A	GAGTGAAGACTTCTAGCGAGGGCCTATTTCCCATGAT
ASAP_BbsI_fwd_B	GAGTGAAGACTTAAAAGAGGGCCTATTTCCCATGAT
ASAP_BbsI_fwd_C	GAGTGAAGACTTGGAAGAGGGCCTATTTCCCATGAT
ASAP_BbsI_fwd_D	GAGTGAAGACTTAAGGGAGGGCCTATTTCCCATGAT
ASAP_BbsI_fwd_E	GAGTGAAGACTTCCAAGAGGGCCTATTTCCCATGAT
ASAP_BbsI_fwd_F	GAGTGAAGACTTAACCGAGGGCCTATTTCCCATGAT
ASAP_BbsI_fwd_G	GAGTGAAGACTTATGCGAGGGCCTATTTCCCATGAT
ASAP_BbsI_fwd_H	GAGTGAAGACTTTTTTGAGGGCCTATTTCCCATGAT
ASAP_BbsI_fwd_I	GAGTGAAGACTTCCCCGAGGGCCTATTTCCCATGAT
ASAP_BbsI_fwd_J	GAGTGAAGACTTGGGGGAGGGCCTATTTCCCATGAT
ASAP_BbsI_fwd_K	GAGTGAAGACTTGGTTGAGGGCCTATTTCCCATGAT
ASAP_BbsI_fwd_L	GAGTGAAGACTTCCTTGAGGGCCTATTTCCCATGAT
ASAP_BbsI_fwd_M	GAGTGAAGACTTTTGGGAGGGCCTATTTCCCATGAT
ASAP_BbsI_fwd_N	GAGTGAAGACTTTTCCGAGGGCCTATTTCCCATGAT
ASAP_BbsI_fwd_O	GAGTGAAGACTTATCGGAGGGCCTATTTCCCATGAT
ASAP_BbsI_fwd_P	GAGTGAAGACTTTAGCGAGGGCCTATTTCCCATGAT
ASAP_BbsI_fwd_Q	GAGTGAAGACTTTACGGAGGGCCTATTTCCCATGAT
ASAP_BbsI_fwd_R	GAGTGAAGACTTCGATGAGGGCCTATTTCCCATGAT
ASAP_BbsI_fwd_S	GAGTGAAGACTTGCATGAGGGCCTATTTCCCATGAT
ASAP_BbsI_fwd_T	GAGTGAAGACTTCGTAGAGGGCCTATTTCCCATGAT
ASAP_BbsI_rev_B	CTCAGAAGACAATTTTGCCATTTGTCTGCAGAATTGG
ASAP_BbsI_rev_C	CTCAGAAGACAATTCCGCCATTTGTCTGCAGAATTGG
ASAP_BbsI_rev_D	CTCAGAAGACAACCTTGCCATTTGTCTGCAGAATTGG
ASAP_BbsI_rev_E	CTCAGAAGACAATTGGGCCATTTGTCTGCAGAATTGG
ASAP_BbsI_rev_F	CTCAGAAGACAAGGTTGCCATTTGTCTGCAGAATTGG
ASAP_BbsI_rev_G	CTCAGAAGACAAGCATGCCATTTGTCTGCAGAATTGG
ASAP_BbsI_rev_H	CTCAGAAGACAAAAAAGCCATTTGTCTGCAGAATTGG
ASAP_BbsI_rev_I	CTCAGAAGACAAGGGGGCCATTTGTCTGCAGAATTGG
ASAP_BbsI_rev_J	CTCAGAAGACAACCCCGCCATTTGTCTGCAGAATTGG
ASAP_BbsI_rev_K	CTCAGAAGACAAAACCGCCATTTGTCTGCAGAATTGG
ASAP_BbsI_rev_L	CTCAGAAGACAAAAGGGCCATTTGTCTGCAGAATTGG
ASAP_BbsI_rev_M	CTCAGAAGACAACCAAGCCATTTGTCTGCAGAATTGG
ASAP_BbsI_rev_N	CTCAGAAGACAAGGAAGCCATTTGTCTGCAGAATTGG
ASAP_BbsI_rev_O	CTCAGAAGACAACGATGCCATTTGTCTGCAGAATTGG
ASAP_BbsI_rev_P	CTCAGAAGACAAGCTAGCCATTTGTCTGCAGAATTGG
ASAP_BbsI_rev_Q	CTCAGAAGACAACGTAGCCATTTGTCTGCAGAATTGG
ASAP_BbsI_rev_R	CTCAGAAGACAAATCGGCCATTTGTCTGCAGAATTGG
ASAP_BbsI_rev_S	CTCAGAAGACAAATGCGCCATTTGTCTGCAGAATTGG
ASAP_BbsI_rev_T	CTCAGAAGACAATACGGCCATTTGTCTGCAGAATTGG
ASAP_BbsI_rev_Z	CTCAGAAGACAACTAGCGCCATTTGTCTGCAGAATTGG
ASAP_BsmBi_fwd_A	GAGTCGTCTCTCCGGTGAGGGCCTATTTCCCATGAT
ASAP_BsmBi_fwd_B	GAGTCGTCTCTAAAAGAGGGCCTATTTCCCATGAT
ASAP_BsmBi_fwd_C	GAGTCGTCTCTGGAAGAGGGCCTATTTCCCATGAT
ASAP_BsmBi_fwd_D	GAGTCGTCTCTAAGGGAGGGCCTATTTCCCATGAT
ASAP_BsmBi_fwd_E	GAGTCGTCTCTCCAAGAGGGCCTATTTCCCATGAT
ASAP_BsmBi_rev_B	CTCACGTCTCATTTTGCCATTTGTCTGCAGAATTGG
ASAP_BsmBi_rev_C	CTCACGTCTCATTCCGCCATTTGTCTGCAGAATTGG
ASAP_BsmBi_rev_D	CTCACGTCTCACCTTGCCATTTGTCTGCAGAATTGG
ASAP_BsmBi_rev_E	CTCACGTCTCATTGGGCCATTTGTCTGCAGAATTGG
ASAP_BsmBi_rev_Z	CTCACGTCTCACCGGTGCCATTTGTCTGCAGAATTGG

To enable Golden Gate cloning into a single TII-RE site in common expression vectors, the first and last TIIS-RE sites of the assembled fragment array are designed to be compatible to the cohesive ends generated by the TII-RE of choice in the destination vector (Fig. 1c). However, this can lead to concatemer formation during restriction-ligation cycles. To prevent this, the insert ends are designed to form a de-novo TII-RE site for an isocaudomer of the TII-RE used to open the destination vector (e.g. XbaI T|CTAGA and NheI G|CTAGC) if ligated to a second insert. The isocaudomer is also added to the reaction and thus simultaneously cleaves any emerging concatemers. Therefore, the one-step assembly and cloning reaction consists of (i) the destination vector, (ii) the insert fragments resulting from the “PCR-on-ligation”, (iii) a TIIS-RE (e.g. BbsI), (iv) a TII-RE for backbone linearization (e.g. XbaI), (v) its isocaudomer for concatemer restriction (e.g. here NheI) as well as (vi) a ligase, and (vii) a specific reaction buffer. This rationally designed combination of reagents ensures that after multiple restriction-ligation cycles, only the desired backbone-insert constructs accumulate (Fig. 1a).

We first demonstrated the feasibility of this approach by introducing multiple GECs into the XbaI site of the pX330 vector. For this purpose, BbsI recognition sites were added to GECs during PCR amplification, and insert concatemers were cleaved by NheI during the subsequent assembly reaction. We successfully integrated up to 9 cassettes simultaneously while keeping a high efficiency of at least 50% positive clones (Fig. 1d). For integrating 10 or more cassettes, we obtained a lower efficiency (below the detection limit) – potentially owing to drastically impaired transformation of larger plasmids (~13 kb with 10 gRNA cassettes). We could show that this system allows to make use of the GEC already encoded on the common pX330 backbone by simply adding a pair of annealed oligonucleotides to the reaction mix (Fig. 1d, lane A and 8 + A). Of note, also reactions in which we omitted NheI yielded the intended vector constructs, although to a markedly reduced efficiency (~58% reduced amount of correct clones). Next, we generated a construct carrying 9 inserted GECs plus the gRNA encoded around the original cloning site, thereby targeting 10 different human genomic loci (“pX330-10 × ”; Supplementary Table 1). After transfection of HEK293T cells with this single vector carrying 10 gRNA expression cassettes, all of the 10 targeted loci displayed indel formation as assessed *via* Surveyor assay or deep sequencing, respectively (Fig. 1e and Supplementary Table 2). Notably, both the improved^7^ and original gRNA scaffolds^10^ used here resulted in similar indel formation efficiencies (Fig. 1f) showing that both scaffolds are suitable for ASAP-cloning-mediated multiplexing in one vector.

**Figure 2:**
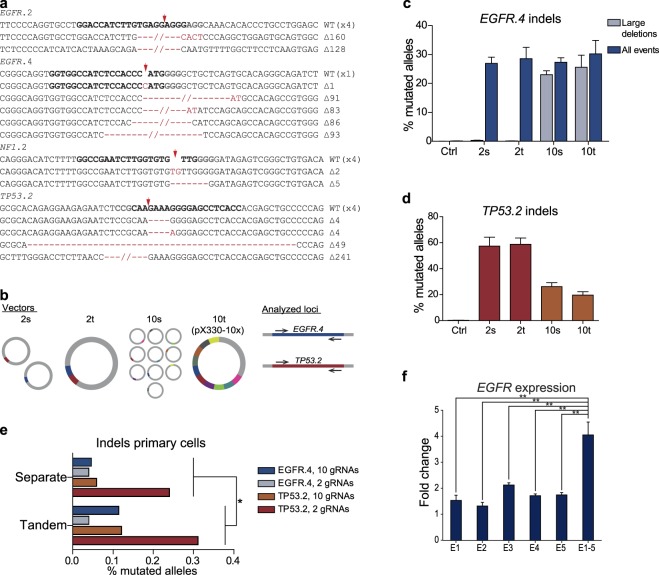
Multiplexed vectors constructed by ASAP cloning outperform single plasmids. (**a**) Sanger sequencing of indicated loci after transfection of HEK293T cells with pX330-10x displays high amount of alleles with frameshifting indels. (**b**) Illustration of the utilized vectors and the analyzed loci of the following experiments. 2 s = Two separate plasmids encoding gRNAs which target the *EGFR*.*4* and the *TP53*.*2* locus, respectively; 2t = single vector encoding these two gRNA cassettes in tandem; 10 s = Ten separate plasmids with ten different genomic targets including the analyzed loci; 10t = One single tandem vector (pX330-10x) encoding gRNAs to target these ten loci. (**c**,**d**) Targeted deep sequencing of the indicated loci after transfection of HEK293T cells with the aforementioned vectors to identify genomic editing events. Deletions encompassing two of the target sites at the *EGFR* locus being 85 bp apart (*EGFR*.*3* and *EGFR*.*4*) were counted and declared as large deletions. Ctrl = untransfected cells; n = 3 (biological replicates). Data are displayed as mean + standard deviation. (**e**) Allele editing events after transfection of primary glioblastoma cells with the indicated, aforementioned vectors. To account for artificial indels at the analyzed loci, respective mutated allele frequencies of WT cells (0.0144% for *TP53*.*2* and 0.07655% for *EGFR*.*4*) were subtracted from mutated allele frequencies of samples. The analyzed loci and the number of delivered gRNAs are depicted in the figure legend. Results of approaches in which separate vectors have been transfected were plotted against tandem approaches. n = 1. P-value ≤ 0.029. The P-value was calculated using a one-sided, paired t-test. (**f**) HEK293T cells were transfected with pcDNA-dCas9-VP64 vectors in which either one separate gRNA expression cassette (E1, E2, E3, E4 or E5) or all together (E1-5) had been integrated *via* ASAP-cloning. The respective gRNAs target the promoter region of *EGFR*. Total RNA was isolated and expression of *EGFR* was assessed by real-time PCR. The graph indicates the fold change of expression compared to cells that had been transfected with an empty vector. P-values: E1 vs. E1-5 ≤ 0.0017, E2 vs. E1-5 ≤ 0.0014, E3 vs. E1-5 ≤ 0.0086, E4 vs. E1-5 ≤ 0.0039, E5 vs. E1-5 ≤ 0.0043. n = 3 (biological replicates). The data are displayed as mean + SEM.

We further confirmed these results by Sanger sequencing of 4 of the 10 loci exemplarily and found that 50% (13/26) of the analyzed alleles displayed Cas9-induced indels (Fig. 2a). Intriguingly, two of the targeted loci within the *EGFR* gene were only 85 bp apart and we detected deletions of the whole intervening DNA segment in four out of six alleles. These deletions also became evident by the occurrence of separate bands after agarose gel electrophoresis of the amplified loci (Supplementary Fig. S1b). This indicates that multiplex gRNA vectors constructed with this method result in a high frequency of deleting larger genomic regions, which is potentially owing to the synchronous expression of both gRNAs in the same cell when delivered on one vector.

As a next step, we aimed at exploiting this concept to elucidate the genome editing efficiencies of Cas9 in the presence of multiple gRNAs. We therefore transfected HEK293T cells with four different vector combinations: (i) two separate vectors targeting the *EGFR* and the *TP53* locus, respectively (2 s), (ii) a vector constructed *via* ASAP-cloning encoding the respective two gRNA cassettes in tandem (2t), (iii) ten separate vectors targeting the two analyzed loci and eight further regions on the genome (10 s), (iv) a single tandem vector to target these ten loci constructed *via* ASAP-cloning (10t; pX330-10x; Fig. 2b).

Transfection of tandem vectors carrying multiple gRNAs resulted in a trend to higher gene editing efficiencies of the *EGFR* locus compared to the transfection of separate vectors, likely owing to higher transfection efficiency (Fig. 2c). Of note, we also found that gene editing efficiencies were independent of the presence of further gRNAs, indicating that a vector delivering 10 gRNAs provides a similar gene editing efficiency for a single locus as a vector delivering only the respective single gRNA. Strikingly, the largest fraction of identified gene editing events comprised the previously identified large deletions (Fig. 2a), again indicating that ASAP-cloning can be used to induce synchronous gene editing, fostering the deletion of the intervening region. The targeted *TP53* locus displayed an overall high percentage of mutated alleles, although editing rates through delivery of 10 RNA expression cassettes were reduced (Fig. 2d). This may, however, be due to larger genomic deletion events induced by two gRNAs, including a target site only 611 bp away from the analyzed *TP53* locus. These larger deletions could not be captured by the PCR product utilized for deep sequencing. We then applied this setup to primary glioblastoma cells that feature major transfection difficulties, resulting in a relatively low overall indel incidence. A detailed analysis of the underlying sequencing reads, however, indicated that the tandem vectors significantly outperformed single gRNA vector mixes (Figs 2e and S2). Thus, delivering multiple gRNA expression cassettes on one vector construct may be generally favorable when targeting more than one locus, particularly in cells that are difficult to transfect.

As a final proof of concept, we sought to demonstrate that this method is applicable also to other vectors and restriction enzyme combinations. Table 2 provides an overview of the utilized backbones and enzymes. We introduced either one or five GECs into the pcDNA-dCas9-VP64 vector commonly used for transcriptional activation. All of the gRNAs targeted a distinct region between 56 bp and 283 bp upstream of the transcriptional start site of the *EGFR* gene to elevate *EGFR* expression. After transfection of HEK293T cells, we found increased *EGFR* expression in all samples. This effect was significantly greater, however, in the cells that had been transfected with the vector carrying five multiplexed gRNA expression cassettes (Fig. 2f).

**Table 2:** Enzymes utilized in ASAP-cloning for multiplex GEC integration into indicated vectors.

Vector	Enzyme cutting inserts	Enzyme cutting vector	Enzyme cutting concatemers (Isocaudomers)
pX330	BbsI	XbaI	NheI
pcDNA-dCas9-VP64	BsmBI	AvaI	AgeI

## Discussion

Over the last decade, the CRISPR/Cas9 system became one of the most popular and versatile tools in the field of molecular biology. To cope with the increasing demand for multiplexed and high throughput genome editing approaches, we here devised a strategy enabling rapid and flexible construction of the required plasmids, termed ASAP-cloning. In comparison to some earlier reports, which were based on distinct destination vectors and required the prior purchase of defined plasmids or plasmid libraries^4–7^, ASAP-cloning allows the utilization of many commonly available vectors as destination backbones, thereby enabling a variety of applications. For example, recently published vectors carrying certain *Cas9* variants or *dCas9* fusions can be used to implement gRNA expression cassettes to efficiently generate “all-in-one” vectors^11,12^. This utility is achieved by a newly devised combination of elaborately constructed primers and a set of restriction enzymes. Besides a TIIS RE, which constitutes the basis of Golden Gate cloning approaches, ASAP-cloning makes use of an enzyme cutting the chosen vector and of a related isocaudomer. This allows to integrate a custom DNA fragment into a common TII-RE site, however, it is a prerequisite that an isocaudomer of the RE opening the vector exists. While this is the case for most common restriction enzymes, it is also important to verify that none of the utilized enzymes cut at unwanted sites within the backbone or any of the inserts. Especially for larger plasmids with many incidental RE sites, finding a fitting set of restriction enzymes may pose a limitation. However, especially for gRNA-expression vectors that don’t encode unwanted sites of the TIIS-RE intended for gRNA cloning, finding a suitable enzyme combination is likely.

Furthermore, so far multiple rounds of cloning were often needed to generate the desired vectors, which required up to 2 weeks of time^4–7^. To circumvent this, ASAP-cloning implements a novel “PCR-on-ligation” step, which allows to complete the entire procedure within a single day. The use of PCR fragments as building blocks for gateway cloning approaches has been described^13^, however, to our knowledge this has not previously been adapted to freshly ligated gRNA expression cassettes and thereby to CRISPR/Cas9-related approaches. Although PCR-products as building blocks for Golden Gate approaches allow saving a tremendous amount of time, it is also important to mention that, in comparison to plasmid libraries, their use requires sequencing of the insert within the final construct due to potential PCR-induced errors. However, this does not necessarily pose a limitation as modern PCR polymerases work with high fidelity making PCR-induced errors extremely rare, and commercially available sequencing services are barely more expensive and time-consuming than control digestions. Furthermore, we could show that the use of an exonuclease and a non-dephosphorylated backbone allows the use of a shortly incubated ligation mix as template for a PCR, which provided the means to generate PCR products of custom gRNA expression cassettes within a few hours. Of note, our system thereby also allows to rapidly generate additional gRNA expression cassettes that can be integrated into previously constructed arrays, providing an unequaled flexibility in comparison to earlier published techniques.

Using ASAP-cloning, we efficiently constructed multiplexed vectors by arraying up to 9 GECs. Attempts to multiplex 10 or more GECs within one approach resulted in a drastically decreased cloning efficiency. This maximum of 9 inserts was in line with earlier publications describing Golden Gate-related cloning techniques^4,14^.

When we assessed Cas9 genome editing efficiencies in the presence of multiple gRNAs, we found that genome editing rates of each analyzed locus remain stable even when up to 9 additional, different gRNAs were present. This indicates that genome editing of multiple loci does not influence the editing potential of a single, given locus within a cell. However, further investigations will be needed to fully elucidate potential limitations of approaches using more that 10 gRNAs or limited amounts of Cas9 protein. Additionally, we could show that genome editing of multiple loci is generally more efficient if the respective gRNAs had been delivered on one vector, highlighting the potential of ASAP-cloning for simultaneous gene editing.

We then performed ASAP-cloning with a plasmid that encodes a Cas9-VP64 fusion construct and gRNAs targeting the *EGFR* promoter to elevate *EGFR* transcription. We found that transcription was most prominently elevated when Cas9-VP64 was recruited to *the EGFR* promoter by multiple gRNAs simultaneously. This is in line with similar findings reported in earlier publications^15–17^. Thus, vector construction provided by ASAP-cloning also allows to robustly and effectively elevate transcription of target genes.

Ultimately, an important characteristic of ASAP-cloning in contrast to previously described techniques is that it is not specifically tailored for CRISPR/Cas9 approaches. The basic strategy that allows arraying of multiple DNA fragments into a common TII-RE site is readily adaptable to other settings. Thus, in the future, ASAP-cloning might also be used for complex cloning approaches such as the combination of genetic elements for transgene expression or the reconstruction of large genes that cannot easily be amplified *via* PCR.

## Methods

### Vector construction by ASAP-cloning

Please refer to the Supplementary Methods section for a detailed ASAP-cloning protocol.

For the construction of a pX330 vector carrying an improved gRNA scaffold^18^ (“pX330-impsc”) the following DNA fragment was synthesized (Integrated DNA Technologies, Leuven, Belgium):

ACATCGTCTCACACCggGTCTTCgaGAAGACctgtttAAGAGCTATGCTGGAAACAGCATAGCAAGTTTAAATAAGGCTAGTCCGTTatcaacttgaaaaagtggcaccgagtcggtgcTTTTTTgttttagagctagaaatagcaagttaaaataaggctagtccgtTTTTagcgcgtgcgccaattctgcagacaaatggctctagaggtacccgttac.

Subsequently, the original pX330 vector was digested with BbsI and KpnI and the synthesized DNA fragment was digested with BsmbI and KpnI. Both fragments were purified *via* the QiaQuick gel extraction kit (Qiagen) and ligated. The resulting vector, pX330-impsc, was utilized in all ASAP-clonings if not stated otherwise.

### Cell culture

HEK293T cells were cultured in IMDM (Thermo Fisher) supplemented with 10% fetal bovine serum (ATCC), 2mM L-Glutamine (Thermo Fisher) and 10 mg/ml penicillin/streptomycin. One day before transfection, 2 × 10_5_ HEK 293 T cells were seeded in six-well dishes containing 2 ml culture medium per well. 2.5 µg of plasmid vector were transfected using Lipofectamine 3000 (Thermo Fisher) according to the manufacturer’s instructions. Three days post transfection, cells were harvested and cell pellets were directly used in subsequent assays or frozen at −20 °C.

### SURVEYOR assay

DNA was isolated with the QiaAMP DNA Mini Kit (Qiagen) at 3 days post transfection of vectors encoding the respective CRISPR nucleases. The potentially disrupted locus was amplified using locus specific primers and PRECISOR polymerase in GC-buffer (BioCat). PCR products were purified using either the QiaQuick gel extraction kit (for PCR products including unspecific products) or the QiaQuick purification kit. Heterodimerization and digestion with SURVEYOR nuclease were performed with the SURVEYOR Mutation Detection Kit (Transgenomic) according to the manufacturer’s instructions. The cleavage products were separated on a 2% agarose gel and stained with ethidium bromide for 10 min. Images were captured with the Gel Doc system (Bio-Rad). Band intensities were quantified *via* ImageJ. Gene modification levels were calculated using the following equation^19^:

$$ \% \,{\rm{genes}}\,{\rm{modified}}=(1-{(1-{\rm{fraction}}{\rm{cleaved}})}^{0.5})\times 100$$

### Sanger sequencing

DNA was isolated as described before. The respective loci were amplified using locus specific primers, which were also utilized in SURVEYOR assays. The PCR products were separated on a 2% agarose gel, stained with ethidium bromide for 10 min and analyzed with the Gel Doc system (Bio-Rad). Subsequently PCR-products of tumor tissue were cloned into pJET1.2 (Thermo Scientific). Plasmids of 6–10 colonies per analyzed locus were subjected to sequencing with an ABI PRISM 7900HT Sequence Detection System (Applied Biosystems). Alternatively sequencing was performed by GATC Biotech, Konstanz, Germany.

### Real-time PCR

RNA was isolated from cell pellets using the RNeasy Mini kit (Qiagen). cDNA was synthesized using SuperScriptII reverse transcriptase (Thermo Fisher) according to the manufacturer’s instructions. The qPCR reaction was performed using SYBR Green (Thermo Fisher). Amplification and signal detection was performed using the ABI PRISM 7900HT System (Applied Biosystems). As housekeeping genes *Gapdh* and *Mldha* were amplified. All samples were measured in three technical replicates. The median CT value of these replicates was used for the following calculation:

$$\frac{{2}^{{\rm{\Delta }}C{T}_{Egfr}(control-sample)}}{{2}^{{\rm{\Delta }}C{T}_{housekeeper}(control-sample)}}=fold\,change$$

Each experiment was performed in biological triplicates, using both housekeeping genes as controls. Thereby, 6-fold changes for each sample were obtained. The mean of these was used as final value. P-values were calculated using the 6 values for fold changes *via* a one-sided t-test assuming unequal variances.

### Targeted deep sequencing

DNA was isolated as described above. The region of interest was PCR-amplified using locus specific primer pairs and PCR products were purified with the QIAquick PCR Purification Kit (Qiagen). After library preparation, barcoded sequencing was performed using the Illumina MiSeq platform, yielding over 1.2 million 251 bp paired-end reads per sample. Reads were aligned to human 1000genomes phase 2 reference genome (hs37d5) combined with phiX174 reference genome (NC_001422.1) using BWA-MEM^20^ (v0.7.8) with –T 0. For downstream analysis we filtered BAM files with SAMtools^21^ (v1.2) using following flags: -F 4 -F 2048 -q 30, which denote mapped reads excluding supplementary alignments, and with mapping quality of at least 30. Extraction of reads corresponding to a given locus and a specific primer pair PCR product within each sequenced sample was based on the leftmost mapping position of reads for all loci except for TP53, which due to primer design and TP53 being on the minus strand required the rightmost mapping position to be calculated from CIGAR string excluding adapter sequences. Finally, insertions and deletions affecting or originating within any of the 6 bases (+/− 3 bp from the Cas9 cut site) were quantified for each locus using a standalone script analyzing CIGAR strings of individual reads, combined with SA tag information in cases where it was present and when the beginning or the end of the read were soft clipped. In those cases, only reads having chimeric segments with mapping quality of 60 were retained. SA tag information was additionally used for quantification of large deletions in cases with two Cas9 cut sites and where both of the sites were affected. Final counting and normalization by total reads per sequenced locus was performed using only non-redundant read names in order to avoid biases due to overlapping mates, so that finally the number of DNA fragments with or without an event was considered.

## Electronic supplementary material

Supplementary Information

## References

[1] Dominguez AA, Lim WA, Qi LS (2016). Beyond editing: repurposing CRISPR-Cas9 for precision genome regulation and interrogation. Nat Rev Mol Cell Biol.

[2] Breunig CT (2018). One step generation of customizable gRNA vectors for multiplex CRISPR approaches through string assembly gRNA cloning (STAgR). PloS one.

[3] Xing HL (2014). A CRISPR/Cas9 toolkit for multiplex genome editing in plants. BMC plant biology.

[4] Sakuma T, Nishikawa A, Kume S, Chayama K, Yamamoto T (2014). Multiplex genome engineering in human cells using all-in-one CRISPR/Cas9 vector system. Scientific reports.

[5] Vad-Nielsen Johan, Lin Lin, Bolund Lars, Nielsen Anders Lade, Luo Yonglun (2016). Golden Gate Assembly of CRISPR gRNA expression array for simultaneously targeting multiple genes. Cellular and Molecular Life Sciences.

[6] Merenda, A. *et al*. A Protocol for Multiple Gene Knockout in Mouse Small Intestinal Organoids Using a CRISPR-concatemer. *Journal of visualized experiments: JoVE*, 10.3791/55916 (2017). 10.3791/55916 PMC5612278 28745625

[7] Lowder LG (2015). A CRISPR/Cas9 Toolbox for Multiplexed Plant Genome Editing and Transcriptional Regulation. Plant Physiology.

[8] Engler C, Kandzia R, Marillonnet S (2008). A one pot, one step, precision cloning method with high throughput capability. PloS one.

[9] Kabadi AM, Ousterout DG, Hilton IB, Gersbach CA (2014). Multiplex CRISPR/Cas9-based genome engineering from a single lentiviral vector. Nucleic acids research.

[10] Cong L (2013). Multiplex Genome Engineering Using CRISPR/Cas Systems. Science.

[11] Hilton IB (2015). Epigenome editing by a CRISPR-Cas9-based acetyltransferase activates genes from promoters and enhancers. Nature biotechnology.

[12] Jinek M (2013). RNA-programmed genome editing in human cells. eLife.

[13] Sanjana NE (2012). A Transcription Activator-Like Effector (TALE) Toolbox for Genome Engineering. Nature protocols.

[14] Engler C, Marillonnet S (2014). Golden Gate cloning. Methods in molecular biology (Clifton, N.J.).

[15] Maeder ML (2013). CRISPR RNA-guided activation of endogenous human genes. Nature methods.

[16] Perez-Pinera P (2013). RNA-guided gene activation by CRISPR-Cas9-based transcription factors. Nature methods.

[17] Cheng AW (2013). Multiplexed activation of endogenous genes by CRISPR-on, an RNA-guided transcriptional activator system. Cell research.

[18] Chen B (2013). Dynamic imaging of genomic loci in living human cells by an optimized CRISPR/Cas system. Cell.

[19] Guschin DY (2010). A rapid and general assay for monitoring endogenous gene modification. Methods in molecular biology (Clifton, N.J.).

[20] Li H (2013). Aligning sequence reads, clone sequences and assembly contigs with BWA-MEM. arXiv.

[21] Li H (2009). The Sequence Alignment/Map format and SAMtools. Bioinformatics (Oxford, England).

