# Supplementary material for: A novel cloning strategy for one-step assembly of multiplex CRISPR vectors
Source: Sci Rep. 2018 Nov 30;8:17499. doi: 10.1038/s41598-018-35727-3 (PMC6269432; doi:10.1038/s41598-018-35727-3)
Supplement: Supplementary file 1 — Supplementary Information [file 41598_2018_35727_MOESM1_ESM.pdf]

# A novel cloning strategy for one-step assembly of multiplex CRISPR vectors

Marc Zuckermann[1,2,*], Mario Hlevnjak[1], Haniyeh Yazdanparast[1], Marc Zapatka[1], David T.W. Jones[2,3,4], Peter Lichter[1,3,*], Jan Gronych[1]

Supplementary Information

# Supplementary Figure S1

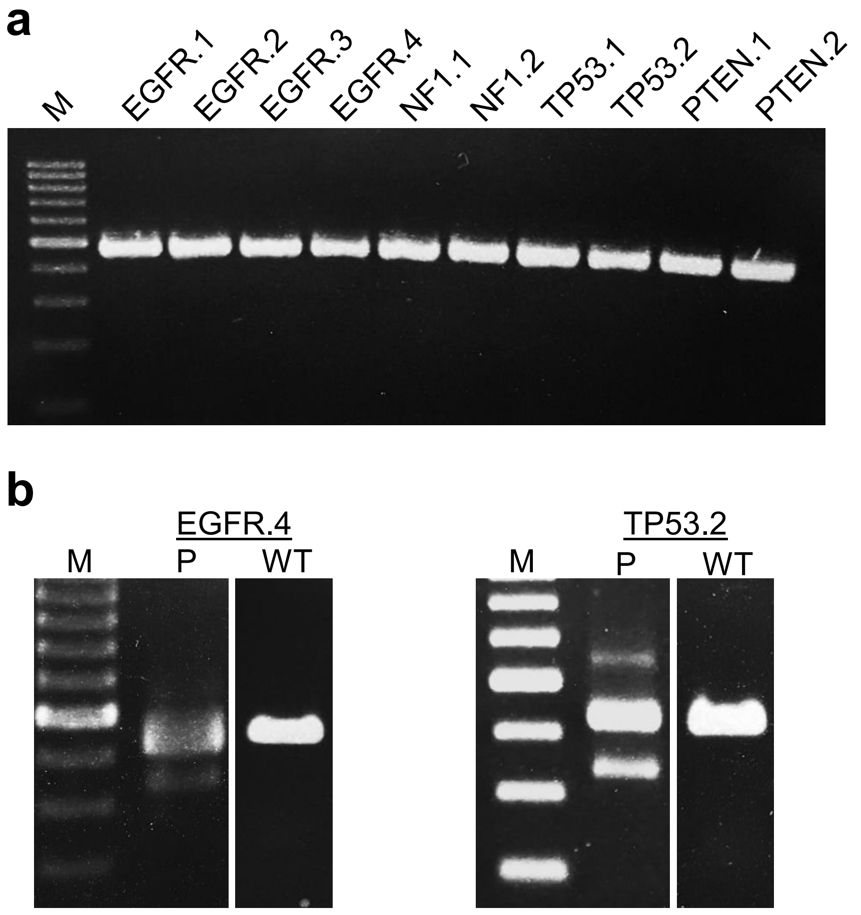


**Supplementary Figure S1** PCR-products. a) Amplified gRNA expression cassettes targeting the indicated loci. b) Uncut PCR-products showing the indicated amplified loci from DNA of cells that have been transfected with pX330-10x (P) or from wild type DNA (WT). M = marker (GeneRuler 100 bp DNA Ladder).

# Supplementary Figure S2

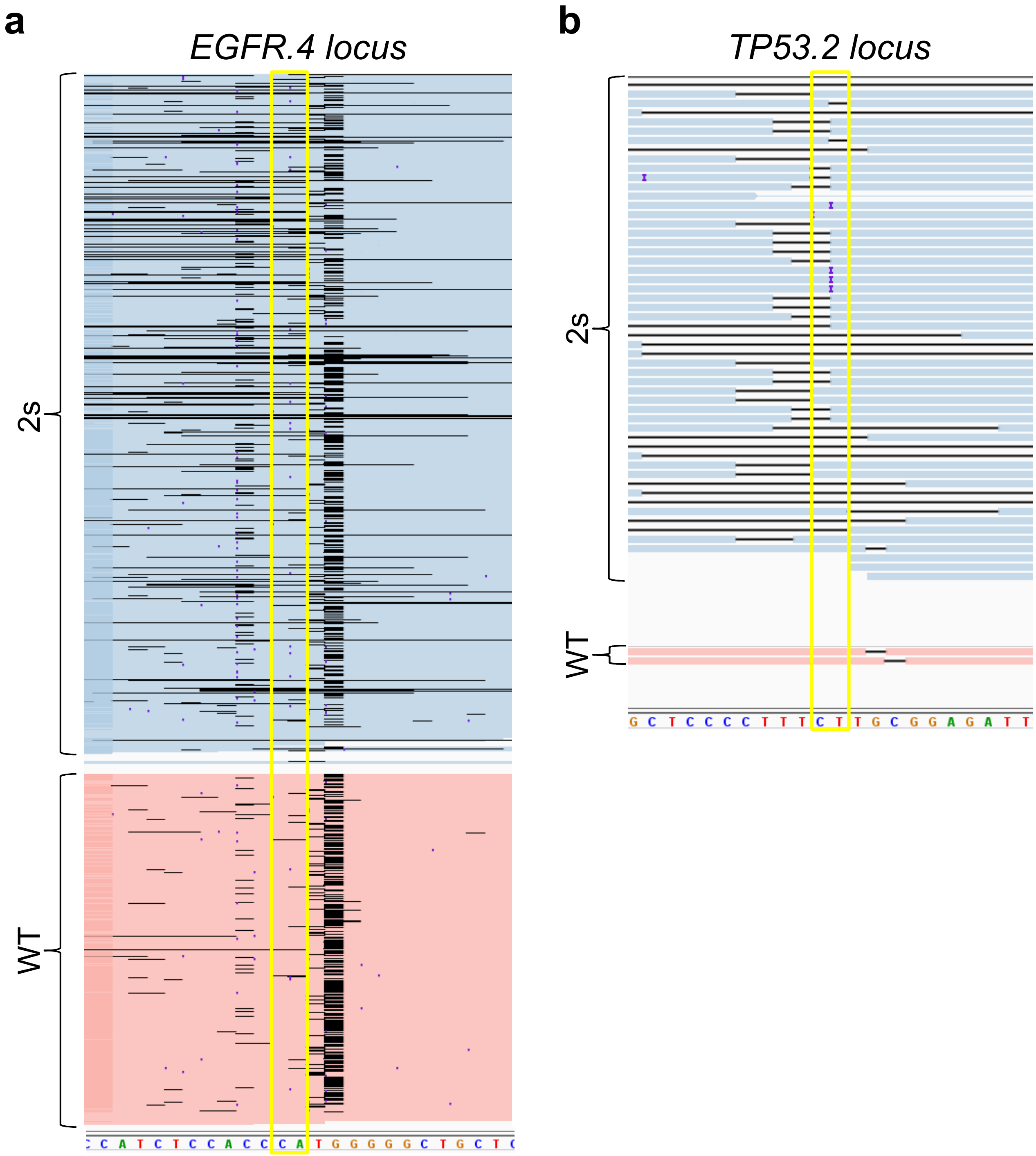


**Supplementary Figure S2** Validation of editing events in primary cells. a),b) Depiction of all reads counted as genome editing events in primary glioblastoma cells. Indicated loci were amplified from DNA of cells that have been untransfected (WT) or transfected with two separate plasmids encoding gRNAs which target the *EGFR.4* and the *TP53.2* locus, respectively (2s). Black, horizontal lines indicate deletions, purple dots indicate insertions. The yellow box indicates the position at which Cas9 cut the DNA. Only indels including at least one of the two bases within the yellow square potentially derived from Cas9-mediated genome editing.

| *Gene* | *CRISPR target sequence* |
|---|---|
| *TP53_1* | GGATGATTTGATGCTGTCCC |
| *TP53_2* | GGTGAGGCTCCCCTTTCTTG |
| *PTEN_1* | GGTTTGATAAGTTCTAGCTG |
| *PTEN_2* | GGTTCATTGTCACTAACATC |
| *NF1_1* | GGCTTGTCGGCAAATCGGGG |
| *NF1_2* | GGCCGAATCTTGGTGTGTTG |
| *EGFR_promoter_1* | GGGTCCAGAGGGGCAGTGCT |
| *EGFR_promoter_2* | ATGCGCCGCCCCACTCCCGC |
| *EGFR_promoter_3* | CCACCGCTGTCCACCGCCTC |
| *EGFR_promoter_4* | CAGAGGAGGAGGAGAATGCG |
| *EGFR_promoter_5* | CCGATCCCTCCTCCGCCGCC |
| *EGFR_1* | GGTATCCAGAACCCCTGGTG |
| *EGFR_2* | GGACCATCTTGTGAGGAGGG |
| *EGFR_3* | GGCTGTGGCTGCTGGATGAG |
| *EGFR_4* | GGTGGCCATCTCCACCCATG |

**Supplementary Table 1** CRISPR target sites.

| *Primer* | *Sequence 5' to 3'* |
|---|---|
| *EGFR_PCR1_fwd* | ACTGAATGCAATCACAAAGGC |
| *EGFR_PCR1_rev* | AAGAGATCCCCATTCCGAGTA |
| *EGFR_PCR2_fwd* | ACTGGCCCATTTCTTGTTTTT |
| *EGFR_PCR2_rev* | GTAGTCCCAGCTACTCAGGGG |
| *EGFR_PCR3_fwd* | TTGCCTTGCTTGGAGGAG |
| *EGFR_PCR3_rev* | CAATTTCCCTAAACCCTGGAG |
| *EGFR_PCR4_fwd* | GAGGGCAAATACAGCTTTGGT |
| *EGFR_PCR4_rev* | CACAAGCACTAAACAGTGGCA |
| *EGFR_qpcr_fwd* | GGGCTCTGGAGGAAAAGAAA |
| *EGFR_qpcr_rev* | GCCCTTCGCACTTCTTACAC |
| *GAPDH_fwd* | GGTGAAGGTCGGAGTCAAC |
| *GAPDH_rev* | CCATGGGTGGAATCATATTG |
| *MLDHA_fwd* | GAAGGACTTGGCGGATGAG |
| *MLDHA_rev* | CCGCGGTGATAATGACCAG |
| *NF1_PCR1_fwd* | GGTCCAGATAATCTCATTTCTCATTTGG |
| *NF1_PCR1_rev* | CCATGTGCTTTGAGGCAGACTGAG |
| *NF1_PCR2_fwd* | GGTTGGTTCTACTGCTGTCCA |
| *NF1_PCR2_rev* | CCCACAACTTGATGAGGTCAC |
| *PTEN_PCR1_fwd* | CTCCTGAATAAAATGGGGGAA |
| *PTEN_PCR1_rev* | GAAATCTAGGGCCTCTTGTGC |
| *PTEN_PCR2_fwd* | TGTTCATCTGCAAAATGGAAT |
| *PTEN_PCR2_rev* | AGTGTCAAAACCCTGTGGATG |
| *TP53_PCR1_fwd* | TTCCTGAAAACAACGTTCTGG |
| *TP53_PCR1_rev* | TCCAAACAAAAGAAATGCAGG |
| *TP53_PCR2_fwd* | GCTAGGAAAGAGGCAAGGAAA |
| *TP53_PCR2_rev* | TCTCCTTACTGCTCCCACTCA |

**Supplementary Table 2** Sequences of further primers used in this study.

Supplementary Methods

**ASAP-cloning protocol**

*The following procedure describes the construction of plasmids harboring multiple gRNA expression cassettes (GECs) using either pX330 or pcDNA-VP64 as vector backbone. The therefore suggested enzymes and oligonucleotides can be found in Table 1 and 2, respectively. Other vector backbones or inserts might require different enzyme combinations and/ or oligonucleotides.*
*In case other vector insert combinations than the ones depicted here are to be used, confirm that backbone and inserts are void of unintended internal recognition sites for the utilized restriction enzymes.*

**Ligation of annealed oligonucleotides (~2 h)**

Annealed oligonucleotides are ligated with BbsI-digested pX330 according to the original Zhang lab protocol (http://www.genome-engineering.org/crispr/wp-content/uploads/2014/05/CRISPR-Reagent-Description-Rev20140509.pdf), except that backbones are not dephosphorylated (by omitting the addition of FastAP during vector digestion), as follows:

1. Digest 1µg of pX330 with BbsI for 30 min at 37°C:

| | |
|---|---|
| 1 µg | pX330 |
| 1 µl | FastDigest BpiI (BbsI; Thermo Fisher) |
| 2 µl | FastDigest Buffer (Thermo Fisher) |
| X µl | dd$H_2O$ |
| 20 µl | total |

2. Add 4 µl DNA Gel Loading Dye (Thermo Fisher), mix and purify the digested vector *via* agarose gel electrophoresis and subsequent gel extraction. Digested pX330 runs around 8.5 kb. Gel extraction can be performed using QIAquick Gel Extraction Kit (Qiagen) or similar.

3. Phosphorylate and anneal each pair of oligonucleotides:

| | |
|---|---|
| 1 µl | oligonucleotide 1 (100µM) |
| 1 µl | oligonucleotide 2 (100µM) |
| 1 µl | T4 Ligation Buffer (NEB) |
| 6.5 µl | dd$H_2O$ |
| 0.5 µl | T4 PNK (5 U; NEB) |
| 10 µl | total |

Anneal in a thermocycler using the following parameters:
37°C for 30 min, 95 °C for 5 min, then ramp down to 25°C at 5°C/min.

4. Set up ligation reaction for each pair of oligonucleotides and incubate at room temperature for 10 min:

| | |
|---|---|
| 50 ng | BbsI digested pX330 from step 2 |
| 1 µl | phosphorylated and annealed oligonucleotides from step 3 (1:250 dilution) |
| 5 µl | Quick ligation Buffer (NEB) |
| X µl | dd$H_2O$ |
| 10 µl | subtotal |
| | |
| 1 µl | Quick Ligase (NEB) |

11 µl total

**“PCR-on-ligation” reaction (~3.5 h)**

5. After ligation, 11 µl of ligation mix are treated with an exonuclease for 30 min at 37 °C:

| | |
|---|---|
| 11 µl | ligation mix |
| 1.5 µl | 25 mM ATP |
| 1.5 µl | 10X Reaction Buffer (Epicentre) |
| 1 µl | Plasmid-Safe DNase (10 U; Epicentre) |

6. Subsequently, the GEC is amplified *via* PCR. Therefore, set up the following reaction on ice in a PCR-tube:

| | |
|---|---|
| 5 µl | GC Buffer (Biocat) |
| 1 µl | dNTP-mix 10mM (Thermo Fisher) |
| 2 µl | Plasmid-Safe treated reaction mix |
| 1 µl | Primer #1 (10µM) |
| 1 µl | Primer #2 (10µM) |
| 0.5 µl | Precisor Polymerase (Biocat) |
| 14.5 µl | $ddH_2O$ |

*The primers used for this PCR must be chosen according to the position of the amplified DNA fragment within the subsequently combined insert, which is to be formed during the assembly reaction. For the examples depicted here, the respective primers can be found in Table 1. These were named alphabetically, whereby a forward primer would always enable binding to a DNA fragment that was amplified using the reverse primer with the same letter. E.g. the first fragment of the insert chain should be amplified using the primers “fwd_A” and “rev_B”, the following fragment with “fwd_B” and “rev_C”, while the last fragment of the array is always amplified with the respective forward primer and “rev_Z”. Oligonucleotides encoding a BbsI site are used for the cloning of GECs into the pX330 vector, whereas primers carrying a BsmBI site can be used to amplify fragments to be inserted into pcDNA-VP64, as this vector harbors internal BbsI sites.*

For the PCR, use the following thermocycler program:

| | | |
|---|---|---|
| 98 °C | 5min | |
| 98 °C | 30sec | 40 cycles |
| 64 °C | 30sec | 40 cycles |
| 72 °C | 20sec | 40 cycles |
| 72 °C | 10min | |
| 4 °C | <1h | |

7. Mix 5 µl of the PCR-product with 1 µl loading dye and separate PCR-products on a 2% agarose gel. For the amplification of gRNA expression cassettes a clear band at 440 bp is to be expected.

***Troubleshooting:*** *If no clear PCR product can be observed, or if unspecific bands or smear arise, the exonuclease treatment might not have worked. In this case control functionality of exonuclease by agarose gel electrophoresis of 100 ng BbsI digested pX330 with and without exonuclease treatment. If the exonuclease proves to be functional, inappropriate PCR conditions might have been the cause. In this case try to optimize the PCR conditions with an intact template before proceeding.*

8. Purify the remaining PCR-product using the QiaQuick PCR purification kit (Qiagen), elute in 50µl $ddH_2O$ and determine the DNA concentration *e.g.* using a NanoDrop device. PCR-products can be frozen for several months at -20°C.

***Troubleshooting:*** *The purified PCR products should have a concentration of about 50 ng/µl. As the utilized backbones for the PCR differ only in the 20 bp protospacer region and are otherwise identical, we encountered little variance for these values. If the quantity of PCR products is low, analyze the functionality of the kit used for PCR purification by purifying a sample with known concentration. If this does not help, increase the reaction volume and reduce the elution volume. To increase the concentration of PCR products for cloning approaches with multiple inserts, inserts can be equimolarly pooled and concentrated by DNA precipitation or by using a PCR purification kit.*

**Assembly reaction (~6 h)**

9. To assemble the DNA fragments into a vector of choice within one reaction the following reaction is set up in a PCR-tube on ice:

| | |
|---|---|
| 1 µl | FastDigest Buffer (Thermo Fisher) |
| 1 µl | Quick ligation buffer (NEB) |
| 1 µl | FastDigest XbaI (pX330) or FastDigest Eco88I (AvaI; pcDNA-VP64) |
| 1 µl | FastDigest NheI (pX330) or FastDigest BshTI (AgeI; pcDNA-VP64) |
| 1 µl | FastDigest BpiI (BbsI; pX330) or FastDigest Esp3I (BsmBI; pcDNA-VP64) |
| 1 µl | Quick ligase (NEB) |
| 30 fmol (~200 ng) | Backbone (pX330 or pcDNA-VP64) |
| 100 fmol (~30 ng) | of each DNA fragment to be inserted |
| up to 20 µl | ddH$_2$O |

To utilize the gRNA expression cassette already encoded on pX330, 1 µl of 10 µM annealed oligonucleotides can be added to the reaction mix. The annealed oligonucleotides will then be inserted into the designated site on the vector during the assembly reaction.
Put the PCR-tube into a thermocycler and run the following program:

| | | |
|---|---|---|
| 37 °C | 20sec | |
| 37 °C | 3min | 26 cycles |
| 16 °C | 4min | 26 cycles |
| 50 °C | 5min | |
| 80 °C | 5min | |
| 4 °C | <1h | |

10. Samples are again treated with an exonuclease for 30 min at 37 °C:

| | |
|---|---|
| 11 µl | Assembly reaction mix |
| 1.5 µl | 25 mM ATP |
| 1.5 µl | 10X Reaction Buffer (Epicentre) |
| 1 µl | Plasmid-Safe DNase (10 U; Epicentre) |

Samples can be frozen for several months at -20 °C.

**Control digestion and sequencing (~2 days)**

11. Subsequently, use the exonuclease reaction mix for bacterial transformation according to the manufacturer's instructions and let the bacteria grow in single clone colonies on agar plates over night.

***Troubleshooting:*** *Using One Shot TOP10 Chemically Competent E. coli (Thermo Fisher) at least 30 colonies can be expected. In case no or few (<5) colonies are observed, analyze the transformation efficiency with an intact plasmid. Using 10 pg of pX330 should result in at least 100 positive transformants. If the transformation efficiency is generally low, optimize before proceeding.*

12. Inoculate 3 ml of growth media containing ampicillin or respective alternative antibiotic with one bacterial colony and incubate at 37 °C over night.

13. Isolate plasmids using a Qiagen Plasmid Miniprep Kit and elute in 50 µl EB. Determine the DNA concentration of each sample *e.g.* using a NanoDrop device.

14. Set up the following reaction and incubate for 1 hour at 37 °C.

| | |
|---|---|
| 2 µl | FastDigest Buffer (Thermo Fisher) |
| 500 ng | Plasmid |
| 1 µl | Enzyme cutting right of the inserted array (e.g. KpnI for pX330 as backone) |
| 1 µl | Enzyme cutting left of the inserted array (e.g. PciI for pX330 as backone) |
| up to 20 µl | dd$H_2O$ |

15. Add 4 µl loading dye to the reaction mix and separate cleavage products on a 1% agarose gel. In case pX330 was used as backbone and cut with KpnI and PciI, a band indicating the backbone around 8.1 kb and a band indicating the inserted array around (440 x (1 + number of inserted GECs)) bp is expected.

***Troubleshooting:*** *We observed that the number of correct clones correlated inversely with the number of inserts that have been utilized in the assembly reaction (Fig. 2c). However, as at least 50% of clones are usually correctly assembled, analyzing a minimum of 4 colonies should suffice to receive the intended vector. If the control digestion indicates an insert of wrong size, make sure the right primers have been used for the respective PCRs.*

16. Vector sequences should be further confirmed *via* Sanger sequencing. As sequencing primers, the same oligonucleotides that were initially annealed and ligated with pX330 to constitute the protospacer complementary region of the gRNA can be used.

***Troubleshooting:*** *If no sequencing results can be obtained, use different primers to sequence the regions of interest. Some of the oligonucleotides initially used to construct the respective gRNAs might possess dismal chemical properties for Sanger sequencing. In case a poor performance for Sanger sequencing is expected for many of the utilized oligonucleotides, the respective regions can be placed at the start or the end of the GEC array. Thereby, those regions can be sequenced using a primer binding the backbone.*
